# In vitro plant regeneration system for date palm (*Phoenix dactylifera* L.): effect of chelated iron sources

**DOI:** 10.1186/s43141-021-00177-4

**Published:** 2021-06-01

**Authors:** Ahmed Madi Waheed Al-Mayahi

**Affiliations:** Date Palm Research Centre, University of Basrah, Basrah, Iraq

**Keywords:** Iron chelate, Shoot regeneration, Antioxidant enzymes, Indole acetic acid (IAA), Rooting

## Abstract

**Background:**

Iron chelate sources and their concentrations are important factors in in vitro propagation of date palm. This study’s objective was to investigate the effect of the iron chelated form on the growth and development of tissue cultures of Barhee cultivar.

**Results:**

The addition of FeEDDHA to the culture medium was more effective than FeEDTA on callus growth, shoot regeneration, and the number of shoots per jar, where the best result (220.8mg callus, 86.67% and 17.2 shoots per jar, respectively) was obtained by using 93.5 mg L^−1^ FeEDDHA (5.6 mg L^−1^ Fe), compared with other treatments. The results also indicate that using 93.5 mg L^−1^ FeEDDHA (5.6 mg L^−1^ Fe) as a supplement can decrease antioxidant enzymes CAT and POD activity compared to the rest of the treatments. Medium equipped with 187.0 mg L^−1^ FeEDDHA (11.2 mg L^−1^Fe) had the highest rooting percentage and number of roots per shoot than other treatments. The biochemical analysis results showed that treatments with FeEDDHA of 280.5 mg L^−1^ (16.8 mg L^−1^ Fe) and 187.0 mg L^−1^ (11.2 mg L^−1^Fe) significantly increased the iron content. The results showed that shoot maximum chlorophyll and endogenous IAA level content were recorded in a medium supplemented with 187.0 mg L^−1^ FeEDDHA (11.2 mg L^−1^Fe) as Fe source.

**Conclusion:**

FeEDDHA used in the present study was proven to be a promising iron chelate source in comparison with the FeEDTA sources.

## Background

Micropropagation is a promising way for obtaining large amounts of uniform plants that are disease-free and without pests during plant material exchange, genetically identical, and high-quality planting material. The increasing demand for rare and excellent quality date palm cultivars makes us use micropropagation as an unavoidable propagation method [1–3]. Plant tissue culture success as a plant propagation method is greatly influenced by the growth media composition [4–6]. Among the micronutrients is iron element. It is an essential element of plant tissue culture media; it is necessary for plant growth and chlorophyll production. Iron (Fe) is critical for many physiological activities like chlorophyll synthesis and biochemical reactions, photosynthesis, respiration, and enzyme activity [7]. This element is involved in the chemical composition of major molecules such as metal-containing enzymes, the Fe-sulfur cluster, the heme, and other Fe-binding sites [8]. To avoid Fe deficiency or toxicity, plants have developed mechanisms to maintain homeostasis by regulating the intake, use, and storage of Fe, depending on its availability for the media [9]. Since Fe is not soluble, particularly in aerobic and high pH conditions, it is rarely accessible to plants. Therefore, it is used more effectively in a Fe chelate form [9]. In in vitro culture conditions, iron is a part of a balanced culture medium that is chemically fixed. Iron is supplied in MS medium [10] as a chelated component of ferric ethylenediaminetetraacetic acid (FeEDTA). This is not a stable form, and Fe released soon becomes unavailable to tissues cultured due to iron-phosphate formation [11]. Iron deficiency or altered pH of the culture medium leads to decreased availability, bud growth inhibition, and reduced chloroplast pigments [12].

Ethylenediamine di-2-hydroxyphenyl acetate ferric (FeEDDHA), due to its high stability and high pH solubility, is the most efficient and used Fe chelates. Commercial FeEDDHA synthesis usually produces multiple position isomers by-products. Replacing ferric ethylenediaminetetraacetic acid (FeEDTA) with an equivalent amount of FeEDDHA had an encouraging effect on micropropagation. Also, FeEDDHA increased the ability to multiply axillary shoots and regenerate adventitious shoots, and induction of roots in the culture medium [13] indicated that the addition of FeEDDHA to the culture medium increased the chlorophyll content compared to FeEDTA. Research on the effect of Fe source on date palm micropropagation is somewhat limited. Therefore, the purpose of this study was to optimize in vitro propagation technique of date palm cv. Barhee, by studying the effect of the chelated form of the iron salt of ferric ethylene di-2-hydroxyphenyl acetate (FeEDDHA) and iron-ethylene diamine-tetracyclic acid (FeEDTA) on callus growth, in vitro regeneration and rooting of shoots, and some of the biochemical parameters.

## Methods

### Plant materials and treatments

The apical buds were sectioned longitudinally into four sections. In order to induce callus formation, explants were transferred to MS basal medium [10] supplemented with 3 mg L^−1^ 6-(dimethylallyl amino) purine (2iP), 30 mg L^−1^ naphthalene acetic acid (NAA), 1.5 g L^−1^ activated charcoal, and solidified with Agar-Agar at 7.0 g L^−1^. Cultures were kept under complete darkness at 27 ± 2 °C. The cultures were transferred to fresh media, with the same composition after every 6 weeks interval until the callus had initiated. For callus propagation, it was transferred and grown in jars containing 25 ml of the MS medium, equipped with 100 mg L^−1^ glutamine, 5 mg L^−1^ thiamine HCl, 1 mg L^−1^ biotin, 30 g L^−1^ sucrose, and solidified with agar at 7.0 g L^−1^ and 0.5 g L^−1^ activated charcoal, with the addition of NAA at 6 mg L^−1^ and 2iP at 2 mg L^−1^. Two Fe forms were included in the medium [FeEDTA (ferric ethylenediaminetetraacetic acid) and FeEDDHA (ethylenediamine di-2-hydroxyphenyl acetate ferric)]. The supplementation of iron sources at different concentrations in the growth medium was assessed. MS medium was modified at two concentrations of FeEDTA [(T1) 36.7 mg L^−1^ (5.6 mg L^−1^ Fe) (control), and (T2) 73.4 mg L^−1^ (11.2 mg L^−1^Fe)] or three concentrations of FeEDDHA [(T3) 93.5 mg L^−1^ (5.6 mg L^−1^ Fe), (T4) 187.0 mg L^−1^ (11.2 mg L^−1^ Fe), and (T5) 280.5 mg L^−1^ (16.8 mg L^−1^ Fe)]. The pH of the medium was adjusted to 5.7–5.8 before the addition of agar. Media dispensed into culture containers. All culture containers with media were autoclaved at 121°C and 1.04 kg.cm^−2^ for 20 min. Cultures were incubated in the culture room at 27 ± 2 °C and irradiated for 16 h with a diffuse light provided by cool white fluorescent lamps (μmol m^−2^ s^−1^). The weight of the callus was recorded after 6 weeks from culturing. For multiplication, the callus was divided and subcultured on regeneration media equipped as mentioned above, except for the plant growth regulators 1 mg L^−1^ (NAA) and 3.0 mg L^−1^ (2iP) [14]. It was also equipped with the same FeEDDHA and FeEDTA concentrations to study their effects on bud multiplication, and some changes in phytochemical traits are mentioned below. Cultures were incubated in the growth chamber at 25 ± 2°C under 16 h photoperiods. The experiments regarding the percentage of bud induction and bud number per jar were recorded after 12 weeks of culturing callus on the multiplication media.

Catalase activity (CAT, EC 1.11.1.6) was assayed, according to [15]. CAT has been verified in the l M of H_2_O_2_ g^−1^ FW min^−1^; the activity of the enzyme has been evaluated at 25± 2 °C. The total 3 mL solution mixture was 2.8 mL (25 mM, pH 7.0), 0.1 mL enzyme extract, and 0.1 mL (0.4%). H 2.8 mL phosphate buffer. Upon adding H_2_O_2_, the reaction began. The decline of H_2_O_2_ inhibition depends on the regulation of absorbance reduction at 240 nm.

### Estimation of peroxidase activity (POD)

Shootlet enzyme extract was prepared as recommended by [16]. The leaf tissues have been grounded with 0.1 M sodium phosphate buffer at pH 7.1 (2 ml buffer/g of fresh tissue in a mortar). These triturated tissues have been strained through four layers of cheesecloth, and the filtrates were centrifuged at 3000 rpm for 20 min at 6 °C. For an estimate, the enzyme of the supernatant fluid was used. The activity of peroxidase was calculated by the methods of [17].

### Effect of FeEDDHA and FeEDTA on in vitro rooting

Clusters of unrooted in vitro shoots of date palm cv. Barhee were collected in the elongation stage. Typical shoots were separated individually and cultured on MS medium [10]. The culture media consisted of MS salts, supplemented with 30 mg L^−1^ sucrose, 0.5 mg L^−1^ NAA, and 0.5 g L^−1^ activated charcoal, 7 mg L^−1^ agar, and different concentrations of FeEDTA (0, 50, 75, and 150 mg L^−1^) or FeEDDHA (0, 0.5, 2.5, and 5.0 mg L^−1^). The pH was adjusted to 5.7–5.8, and then, the media were autoclaved at 121°C for 20 min. All cultures were incubated under room temperature 25 ± 2 °C, with a 16 h photoperiod provided by white florescent light. The experiment results regarding the percentage of root induction and root number per shoot were evaluated 6 weeks after the inoculation of shoots on the culture media.

### Mineral analysis of shoots

Elemental analysis was performed on content of potassium (K), calcium (Ca), and boron (B) in date palm shoots after 12 weeks of culture according to the method described by [18]. Before the analysis, the shoots were separated and washed with deionized water twice and dried at 60°C until reaching constant weight. Dried shoots (0.5 g) were digested with a mixture of sulfuric-perchloric acid under heating for 1 h. The digested solution was transferred into volumetric flask 50 cm^3^, and volumes were completed in size with distilled water. The extract of the samples was filtered and diluted to the volume of 50 ml. Ca and Mg content was determined by flame atomic absorption spectrophotometry. A UV-visible spectrophotometer measured the absorbance of the solution at a wavelength of 620 nm [19]. For B analysis, dried shoots were ash dried (60 °C for 1 h) and digested with 10 ml 0.36 N H_2_SO_4_ [20]. B was quantified by a spectrometer. There were three replicates of each treatment. The atomic absorption spectrophotometer determined the shoot samples’ iron content at the wave length of 324.8 nm.

#### Assessment of chlorophyll content

The chlorophyll content in the leaves was measured using the method of [21].

#### Extraction and measurement of auxins

Auxins were extracted and quantified according to [22]. Five grams of leaves after various treatments with Fe-EDDHA and FeEDTA were homogenized using 80% methanol. The extract has been filtered through the Whatman filter paper (no. 1) and evaporated at 4 °C in dark conditions under a vacuum. The supernatant was dried in a vacuum, withdrawn by a 0.1 M phosphate potassium (pH 8.1). Eleuate was obtained by using 1 N hydrochloric acid (HCI) and by using a partitioning (4x) with diethyl ether, in dryness, in water with a pH set to 2.5. The injection in reversed HPLC, C18 column, in the isocratic elution mode by the concentrate, determined phytohormones using a portable acetone step (26:74) with 30 mM of phosphoric acids. A UV detector (2996 PDA detector) with 280 nm was passed through the column eluants, and auxins were detected and quantified. Standard auxins were used as the source (IAA).

### Experimental design and statistical analysis

The data were statistically analyzed by one-way analysis of variance (ANOVA) using statistical analyses with the SPSS packet software. Separation of means among treatments was determined using L.S.D. test at 5%.

## Results

**Fig. 1:**
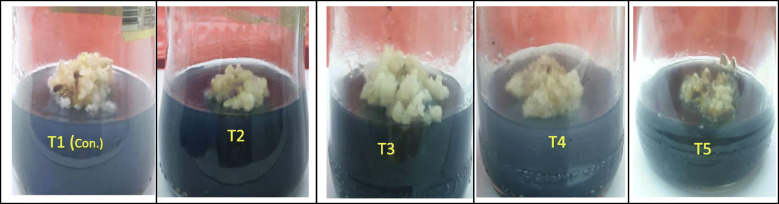
Callus growth on MS media supplemented with chelated iron sources after 12-week treatment (T1) 36.7 mg L^**−**1^ FeEDTA (5.6 mg L^**−**1^ Fe) (control), (T2) 73.4 mg L^−1^ FeEDTA (11.2 mg L^−1^Fe), (T3) 93.5 mg L^**−**1^ FeEDDHA (5.6 mg L^**−**1^ Fe), (T4) 187.0 mg L^−1^ FeEDDHA (11.2 mg L^−1^Fe), and (T5) 280.5 mg L^**−**1^ FeEDDHA (16.8 mg L^−1^ Fe)

**Fig. 2:**
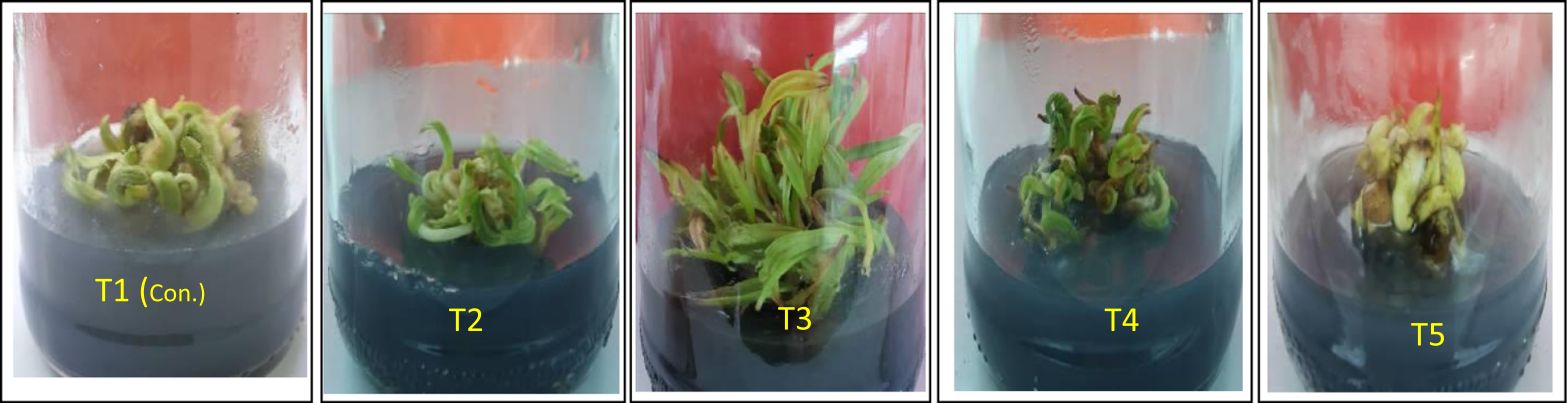
Bud induction on MS media supplemented with chelated iron sources (T1) 36.7 mg L^−1^ FeEDTA (5.6 mg L^−1^ Fe) (control), (T2) 73.4 mg L^−1^ FeEDTA (11.2 mg L^−1^Fe), (T3) 93.5 mg L^−1^ FeEDDHA (5.6 mg L^−1^ Fe), (T4) 187.0 mg L^−1^ FeEDDHA (11.2 mg L^−1^Fe), and (T5) 280.5 mg L^−1^ FeEDDHA (16.8 mg L^−1^ Fe)

The results from the present study indicate that the callus tissues grown at FeEDDHA (93.5 mg L^−1^) (5.6 mg L^−1^ Fe) showed better growth (220.8 mg100/mg callus) (Table 1) followed by a high induction of buds with the highest average of shoot formation (Fig. 2, T3), which were 86.67%, 17.2 shoots, respectively. On the other hand, callus weight, shoot multiplication, and average shoot formation significantly decreased at double FeEDTA and triple FeEDDHA concentration in MS medium (Table 1, Figs. 1 and 2, T2 and T5).

**Table 1:** Effect of chelated iron sources on callus growth, a response percentage (%) of callus for bud formation, and the number of buds

Treatments (mg.L^−1^)	Callus growth	Response of callus for buds regeneration (%)	Mean number of buds/100 mg
**T1 (control)**	**198.2±1.79 b**	**66.67±3.05c**	**12.2±0.25b**
**T2**	**141.0±3.60 c**	**53.34±2.60d**	**7.6±0.38c**
**T3**	**220.8±2.21a**	**86.67±6.88a**	**17.2±0.83a**
**T4**	**187.0±2.05 b**	**73.34±4.81bc**	**10.0±0.38b**
**T5**	**116.4± 2.44 d**	**46.67±4.70d**	**6.0±0.21c**

Means in each column with different letters show significant differences (*P* ≤ 0.05 )

### The activity of catalase (CAT) and peroxidase (POD)

**Fig. 3:**
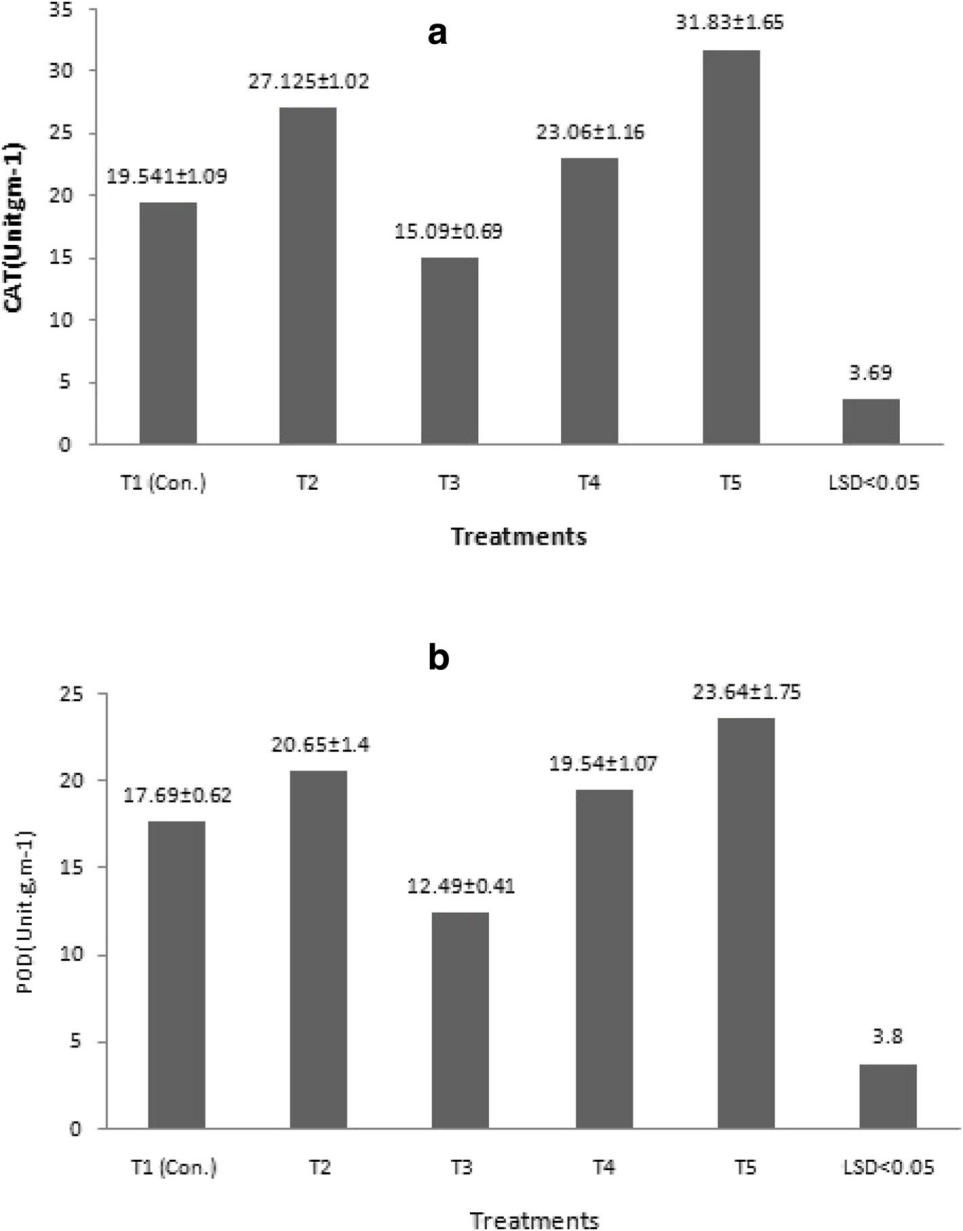
Effect of chelated iron sources FeEDTA and FeEDDHA on **a** catalase (CAT) and **b** peroxidase (POD) activity in shoots

The highest CAT and POD were obtained when callus was cultured in the medium supplied with a high concentration of FeEDDHA 280.5 mg L^−1^ (16.8 mg L^−1^ Fe) (Fig. 3, T5), significantly different from the other treatments. Simultaneously, the least CAT and POD activity of the shoots was achieved in the medium supplied with 93.5 mg L^−1^ FeEDDHA (5.6 mg L^−1^ Fe) (Fig. 3A and B).

### Effect of Fe sources on the rooting formation

**Fig. 4:**
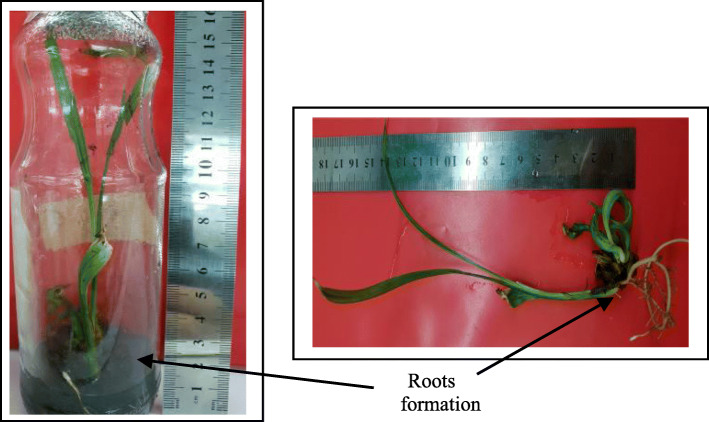
Rooting of date palm shoot cv. Barhee on MS medium supplemented with 187.0 mg L^−1^ FeEDDHA (11.2 mg L^−1^Fe), after 45 days from shoot culture on the rooting medium

According to the results obtained in Fig. 1 (Table 2), increasing FeEDDHA concentration of the medium from 93.5 (5.6 mg L^−1^ Fe) to 187.0 mg L^−1^ (11.2 mg L^−1^Fe) resulted in a percentage of rooting increasing and the number of roots. The result was different from FeEDTA when its concentration was increased from 36.7 (control) to 73.4 mg L^−1^ (11.2 mg L^−1^ Fe). However, the 187.0 mg L^−1^ FeEDDHA application gave the highest response percentage of shoots producing roots with the highest number of roots (Fig. 4) compared with the other treatments after 6 weeks from shoot culture, while the 280.5 mg L^−1^ FeEDDHA application gave the highest length of roots.

**Table 2:** Effect of chelated iron sources FeEDTA and FeEDDHA on in vitro rooting

Treatments (mg.L ^−1^)	Response shoots for root regeneration (%)	Mean number of roots/shoot	Length (cm)
**T1 (control)**	**66.67±4.33cd**	**4.1± 0.39 b**	**1.01±0.2cd**
**T2**	**60.00±4.90d**	**3.6±0.30bc**	**0.90±0.21d**
**T3**	**80.00±3.89ab**	**5.7± 0.23 a**	**2.27±0.47b**
**T4**	**86.67±6.40a**	**6.1± 0.81 a**	**3.90±0.42a**
**T5**	**73.34±3.39bc**	**2.8± 0.25 c**	**4.70±0.23a**

Means in each column with different letters show significant differences (*P* ≤ 0.05)

### Effect of Fe sources on some biochemical traits

#### Mineral content

The chelated iron source treatment led to significant effects on the accumulation of iron (Fe) in the date palm shoot tissues. Shoots grown in medium supplement with 93.5 mg L^−1^ FeEDDHA (5.6 mg L^−1^ Fe) induced significant increases in shoot tissue Fe (24.8 %) when compared with the other treatments (Table 3). No significant effect of chelated iron sources on shoot nutrient content was shown regarding magnesium (Mg), calcium (Ca), as well as boron (B) (Table 3).

**Table 3:** Effect of chelated iron sources FeEDTA and FeEDDHA on the nutrient content in the shoots of date palm cv. Barhee in vitro

Treatments (mg.L^−1^)	Mg (mg g^**−1**^ DW.)	Ca (mg g^**−1**^ DW.)	B (μg^**–1**^ DW)	Fe (μg^**–1**^ DW)
**T1 (control)**	**13.174±0.41a**	**18.619±0.87a**	**2.088±0.5a**	**0.298±0.13d**
**T2**	**13.153±0.50a**	**18.779±0.88a**	**2.350±0.4a**	**0.528±0.08c**
**T3**	**13.986±0.95a**	**19.399±0.52a**	**2.914±0.3a**	**0.783±0.06b**
**T4**	**14.068±0.66a**	**19.582±0.47a**	**3.010±0.2a**	**1.013±0.04a**
**T5**	**13.744±0.65a**	**18.800±0.87a**	**2.694±0.2a**	**1.110±0.05a**

Means in each column with different letters show significant differences (*P* ≤ 0.05)

#### Changes in chlorophyll

The chelated iron source treatment led to significant effects on the accumulation of iron (Fe) in the date palm shoot tissues. Shoots grown in medium supplement with 280.5 mg L^−1^ FeEDDHA (16.8 mg L^−1^ Fe) induced increases in shoot tissue Fe (1.110) when compared with the other treatments (Table 4). No significant effect of chelated iron sources on shoot nutrient content was shown regarding magnesium (Mg), calcium (Ca), as well as boron (B) (Table 4).

**Table 4:** Effect of chelated iron sources FeEDTA and FeEDDHA on the content of chlorophylls a, b, and total chl in the date palm cv. Barhee in vitro

Treatments (mg.L^**−1**^)	Chl a (mg g^**−**1^ FW)	Chl b (mg g^**−**1^ FW)	Total Chl (mg g^**−**1^ FW)
**T1 (control)**	**0.72±0.023 d**	**0.099±.004d**	**0. 819±0.019d**
**T2**	**0.92± 0.04 c**	**0.130±0.002c**	**1.05±0.0016 c**
**T3**	**1.12±0.07 b**	**0.177±0.08b**	**1.297±0.061b**
**T4**	**1.39± 0.10 a**	**0.243±0.003a**	**1.633±0.08a**
**T5**	**1.01± 0.06 bc**	**0.149±0.002c**	**1.159±0.20bc**

Means in each column with different letters show significant differences (*P* ≤ 0.05)

#### Endogenous IAA levels

**Fig. 5:**
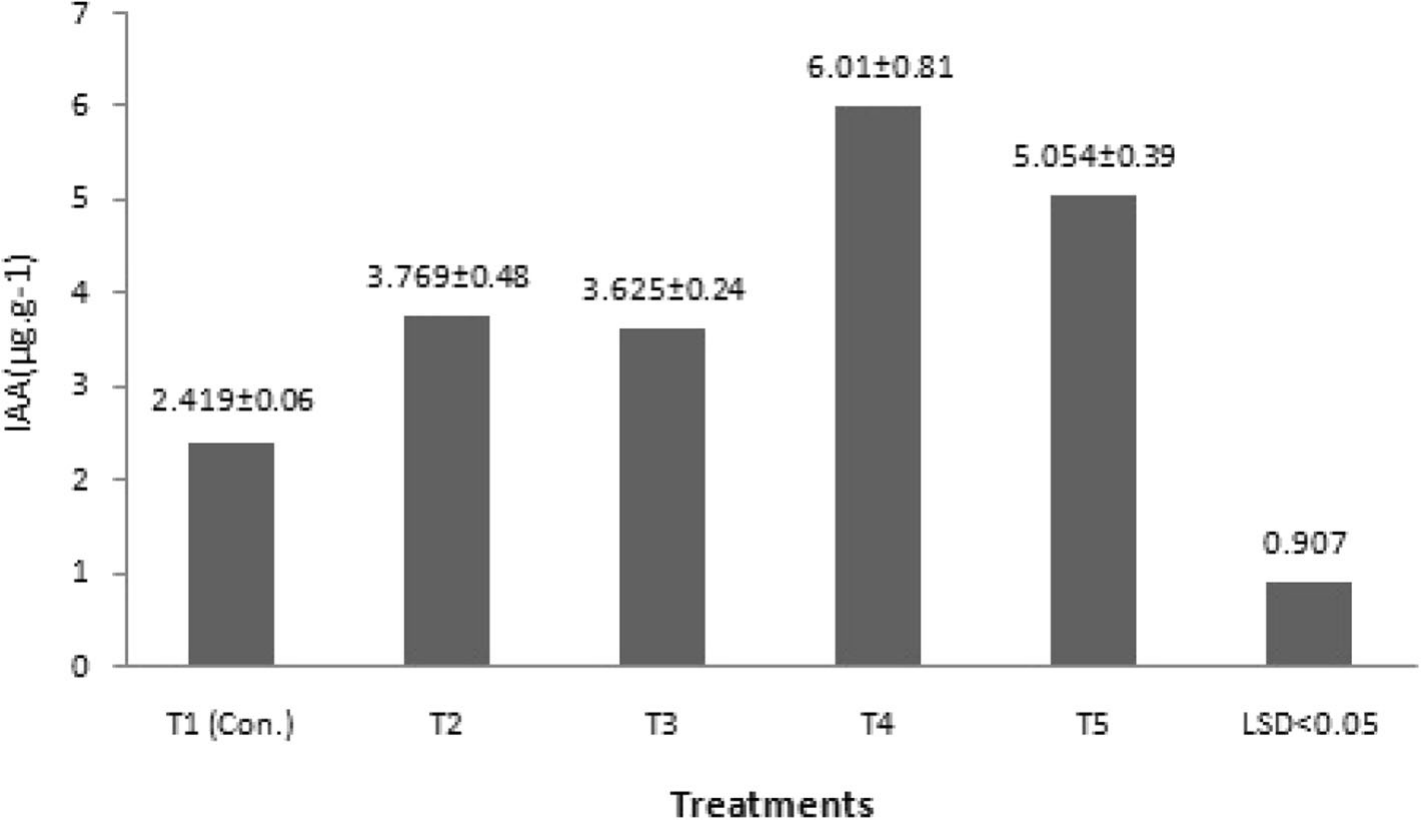
Effect of chelated iron sources FeEDTA and FeEDDHA on endogenous IAA content

Figure 5 shows the effect of FeEDTA and FeEDDHA on endogenous IAA content. Shoots cultured in medium supplement with 187.0 mg L^−1^ FeEDDHA (11.2 mg L^−1^ Fe) showed the highest IAA content (6.10 μg.gm^−1^) which was significantly different than what was reported at the shoots grown in the other media (*p*<0.05). On the other hand, the lowest contents were recorded in shoots grown in the medium supplemented with FeEDTA at 36.7 mg L^−1^ (5.6 mg L^−1^ Fe) (control).

## Discussion

The results showed that iron sources and their concentrations are important factors in propagating the date palm cv. Barhee in vitro. The addition of 93.5 mg L^−1^ FeEDDHA (5.6 mg L^−1^ Fe) to MS medium resulted in better growth measured by increased callus weight, response percentage, and a number of shoots per jar (220.8 mg, 86.87%, and 17.3 buds) respectively, which can be recommended for callus growth and shoot multiplication. FeEDTA, typically used as a source of iron in the media, is unstable in light, and iron rapidly becomes unavailable to cultures. More than 75% of the total iron is consumed by the tissue cultured in the first week of culturing [23]. Although the medium containing FeEDDHA was essential for callus growth and *P. dactylifera* L propagation, the results showed high concentrations of FeEDDHA-inhibited callus growth, development, and bud proliferation. We observed inhibition of shoot multiplication on culture media equipped with a double and triple concentration of FeEDDHA in growth media (Table 1). Microelement salt concentration can play a significant role in the micropropagation of woody plants [24, 25]. The addition of FeEDDHA to the culture medium increased the percentage of leaf formation in some red raspberry cultivars and the number of adventitious buds in all five studied cultivars [26]. FeEDDHA beneficial effect on in vitro cultured tissues’ high stability is attributed to the high stability of this type of iron chelate, which enables iron to be obtained and maintain a stable ionic balance in the culture medium [27]. In consistency with our experiment results, the beneficial effect of FeEDDHA on adventitious buds induction was confirmed by [28] for many blackberry cultivars. Iron (Fe) is one of the micronutrients; it is essential for plant growth and chlorophyll production. Fe is necessary for many physiological activities like chlorophyll synthesis and biochemical reactions, photosynthesis, respiration, and enzyme activity [7]. The high levels of FeEDDHA showed the maximum CAT and POD activity. The increased activity of CAT and POD in the control treatment and FeEDDHA at high concentrations may be interpreted as an attempt by date palm plants to overcome ROS accumulation under iron deficiency or excess conditions compared to other treatments.

A prerequisite for any method of propagation is the effective rooting of in vitro shoots before their establishment in the soil [29, 30]. To our knowledge, this is the first study of using Fe-EDDHA on in vitro propagation of date palm, including rooting. However, in other plant species like apple [31], reported that the highest root number in ½ MS + 0.15 g/l Fe-EDDHA + 2.4 g/l thiamin. FeEDDHA stimulating effect on rooting has been reported in many plants, such as Prunus GF-677 and citrus rootstocks [32, 33].

Iron (Fe) is believed to have a role in protein synthesis, root development, and a lack of nitrates in plants that accumulate due to ferredoxin. At the appropriate concentration and the optimal chemical type on in vitro rooting, the effect of iron may be correlated with peroxidase and catalase enzyme activities, which both affect auxin metabolism [34]. FeEDDHA, which is most stable, does not interfere with auxin metabolism and does not produce reactive oxygen species [35]. Altering FeEDTA to FeEDDHA in the medium increases iron availability in many plant species and reduces micronutrient deficiency [36] .

In light of our findings, replacing FeEDTA with FeEDDHA and increasing the concentration of iron ions in the culture medium resulted in better plantlet quality, with an increase in chlorophyll content in shoots in the presence of FeEDDHA.

Iron deficiency inhibits the growth of chloroplasts in plant leaves. It is essential for chlorophyll formation and plant development. This factor is an important part of the energy plants [37]. Shoots grown in a medium supplemented with the Fe-EDDHA had more amount of chlorophyll than Fe-EDTA. These results showed the efficacy of using Fe-EDDHA in culture media to improve the date palm micropropagation efficiency.

Auxin affects a wide range of growth and development processes in the plant, including cell elongation, cell division, and rooting. Auxin is involved in plant organogenesis. EDTA may stimulate IAA action by suppressing its degradation through IAA decarboxylation [38]. Additionally, auxin is one of the essential plant hormone classes for the growth and development of in vitro organs. Under different stress conditions of iron concentrations, endogenous IAA accumulation was higher in *M. baccata* roots at lower levels compared to higher levels [39]. These observations confirm our results that increased cellular auxin levels cause enhanced root formation [40].

## Conclusion

Media supplemented with 93.5 mg L^−1^ (5.6 mg L^−1^ Fe) FeEDDHA gave the highest callus induction and bud formation. Additionally, CAT and POD activity increased in in vitro shoots regenerated in the same medium mentioned above. Significant increases in rooting percentage and root number were also observed on shoots grown in a medium containing FeEDDHA 187.0 mg L^−1^ (11.2 mg L^−1^ Fe) compared with other treatments. Furthermore, FeEDDHA allows a substantial increase of endogenous hormone levels (IAA) and chlorophyll in shoots. Accordingly, this increase can affect root development in shoots of date palm cv. Barhee. Therefore, we suggest applying MS medium with 93.5 mg L^−1^ (5.6 mg L^−1^ Fe) FeEDDHA for the callus and multiplication phase, followed by rooting with 187.0 mg L^−1^ (11.2 mg L^−1^ Fe).

## Data Availability

All data generated or analyzed during this study are included in this article.

## Abbreviations

NAA: Naphthaleneacetic acid
IAA: Indoleacetic acid
2iP: N6-(2-Isopentenyl) adenine
FeEDTA: Ferric ethylenediaminetetraacetic acid
FeEDDHA: Ethylenediamine di-2-hydroxyphenyl acetate ferric
CAT: Catalase
POD: Peroxidase
